# Estimating the Exposure–Response Relationships between Particulate Matter and Mortality within the APHEA Multicity Project

**DOI:** 10.1289/ehp.7387

**Published:** 2004-10-21

**Authors:** Evangelia Samoli, Antonis Analitis, Giota Touloumi, Joel Schwartz, Hugh R. Anderson, Jordi Sunyer, Luigi Bisanti, Denis Zmirou, Judith M. Vonk, Juha Pekkanen, Pat Goodman, Anna Paldy, Christian Schindler, Klea Katsouyanni

**Affiliations:** ^1^Department of Hygiene and Epidemiology, University of Athens, Athens, Greece; ^2^Harvard School of Public Health, Boston, Massachusetts, USA; ^3^Community Health Sciences, St. George’s Hospital Medical School, University of London, London, United Kingdom; ^4^Institut Municipal Investigacio Medica (IMIM), Barcelona, Spain; ^5^Azienda Sanitaria Locale della Città di Milano, Milano, Italy; ^6^INSERM U420, Nancy, France; ^7^Department of Epidemiology and Statistics, University of Groningen, Groningen, the Netherlands; ^8^National Public Health Institute, Unit of Environmental Epidemiology, Kuopio, Finland; ^9^Dublin Institute of Technology, Dublin, Ireland; ^10^National Institute of Environmental Health, Budapest, Hungary; ^11^University of Basel, Institut fur Sozial-und Praventivmedizin, Basel, Switzerland

**Keywords:** air pollution, exposure–response, heterogeneity, hierarchical modeling, mortality, splines

## Abstract

Several studies have reported significant health effects of air pollution even at low levels of air pollutants, but in most of theses studies linear nonthreshold relations were assumed. We investigated the exposure–response association between ambient particles and mortality in the 22 European cities participating in the APHEA (Air Pollution and Health—A European Approach) project, which is the largest available European database. We estimated the exposure–response curves using regression spline models with two knots and then combined the individual city estimates of the spline to get an overall exposure–response relationship. To further explore the heterogeneity in the observed city-specific exposure–response associations, we investigated several city descriptive variables as potential effect modifiers that could alter the shape of the curve. We conclude that the association between ambient particles and mortality in the cities included in the present analysis, and in the range of the pollutant common in all analyzed cities, could be adequately estimated using the linear model. Our results confirm those previously reported in Europe and the United States. The heterogeneity found in the different city-specific relations reflects real effect modification, which can be explained partly by factors characterizing the air pollution mix, climate, and the health of the population.

Many epidemiologic studies in recent years have documented adverse effects of ambient particulate matter (PM) concentrations on mortality (Katsouyanni et al. 2001; Pope et al. 1995; Samet et al. 2000). The indications of adverse health effects even at below-guideline levels have led to revisions of air quality guidelines and standards and scheduled dates for regular revisions in the future [Commission of European Communities 1999; U.S. Environmental Protection Agency (EPA) 1996; World Health Organization (WHO) 2000]. Most of these studies have assumed linear associations between air pollution and daily deaths, although in cases where concentrations reached high levels, logarithmic transformations have frequently been used (Touloumi et al. 1994). However, the shape of the exposure–response relationship is crucial for public health assessment, and there has been growing demand for providing the relevant curves. Whether or not there is a threshold makes a large difference to the estimate of attributable deaths, and the shape of the exposure–response association is important for predicting the benefits of policies reducing exposure.

Recently, multicity national or international programs have provided results based on data from many cities (Katsouyanni et al. 2001; Samet et al. 2000). Combined evidence was obtained using hierarchical models implemented in two stages. In the first stage, data from each city were analyzed separately, whereas in the second stage, the city-specific air pollution estimates were regressed on city-specific covariates to obtain overall estimates and to explore sources of possible heterogeneity.

In the United States, several multicity studies have explored the exposure–response association between particulate air pollution and mortality (Daniels et al. 2000; Dominici et al. 2002a; Schwartz and Zanobetti 2000). A linear association without threshold was seen. Particulate characteristics differ considerably between Europe and the United States, and the high penetration of diesel engines in Europe makes mobile sources a much more important source of urban particles there. Schwartz et al. (2001) confirmed that the exposure–response relation between airborne particles and total daily deaths is essentially linear, at least at low to moderate concentrations in eight cities in Spain. Similarly, Rossi et al. (1999) found that in Milan, Italy, the association for all causes and cause-specific deaths was almost identical to that noted by Schwartz et al. (2001).

One key limitation of these European studies (Rossi et al. 1999; Schwartz et al. 2001) has been the use of data from a single or a few locations. We address this limitation by presenting the results of analyses examining the exposure–response relationship between daily deaths and airborne particles within the APHEA-2 (Air Pollution and Health—A European Approach) project (Short-Term Effects of Air Pollution on Health: A European Approach to Methodology, Exposure–Response Assessment and Evaluation of Public Health Significance) that uses an extensive European database from 30 cities. This database also allows a comprehensive and structured approach at the second stage of the analysis, in which we explore the role of effect modifiers in explaining the heterogeneity in the shape of the exposure–response relation of air pollution and mortality across cities.

## Materials and Methods

### Data.

The APHEA-2 project was a multicenter study including 30 cities across Europe and associated regions (i.e., Istanbul, Turkey, and Tel Aviv, Israel) that studied health effects of air pollution. Data were collected on daily counts of all-cause mortality (excluding deaths from external causes) [*International Classification of Diseases*, *9th Revision* (ICD-9; WHO 2002) codes > 800], cardiovascular mortality (ICD-9 390–459), and respiratory mortality (ICD-9 460–519). The data covered at least 3 consecutive years for each city within the years 1990–1997. Details about the data have been published elsewhere (Katsouyanni et al. 2001).

Daily air pollution measurements were provided by the monitoring networks established in each town participating in the APHEA-2 project. A monitor was included if certain completeness criteria were fulfilled (Katsouyanni et al. 1996). Time-series data on daily temperature (degrees centigrade, daily mean) and relative humidity (percent) were used to control for the potential confounding effects of weather. External information on influenza epidemics or other unusual events (e.g., heat waves, strikes) was also collected, if available (Katsouyanni et al. 2001).

In the present study we used the average of lags of 0 and 1 day for black smoke (BS) and PM < 10 μm in aerodynamic diameter (PM_10_) for all cities, because there is evidence that the average of 2 days’ pollution correlates better with mortality than a single day’s exposure (Schwartz 2000a).

Table 1 presents descriptive characteristics of the analyzed cities. The Netherlands is considered one urban area because of its relatively small size and dense population. Together, all 30 areas have a population of > 60 million people. The mean daily total number of deaths ranged from 6 (in Erfurt and Geneva) to 342 in the Netherlands. For respiratory mortality, daily rates ranged from 0 to 29. The median levels of BS and PM_10_ concentrations ranged from 9 to 63 μg/m^3^ and from 14 to 65 μg/m^3^, respectively. BS levels represent concentrations of black particles with an aerodynamic diameter < 4.5 μm (Department of Health 1995). These measurements have a long history in Europe, and although standards for BS have been replaced recently by those for PM_10_ (Commission of European Communities 1999), the results are displayed here both for continuity and because there is evidence that BS exposure is more relevant to health effects than is PM_10_ (Bremner et al. 1999; Brunekreef et al. 1997). BS is a better marker of primary combustion products and small particles (Reponen et al. 1996). Because domestic or industrial burning of coal is minimal in most of the cities studied, BS is more specific for traffic-related particles than PM_10_ and provides a means of addressing the question of particle composition.

### Methods.

We used a hierarchic modeling approach. First, we fit regression models in each city separately to control for potential confounders. We used the results of the individual city analysis in a second-stage analysis to provide overall estimates and to investigate potential effect modifiers.

### Individual city analysis.

We investigated the pollution–mortality associations for each city using Poisson regression models allowing for overdispersion. The city-specific model is of the form


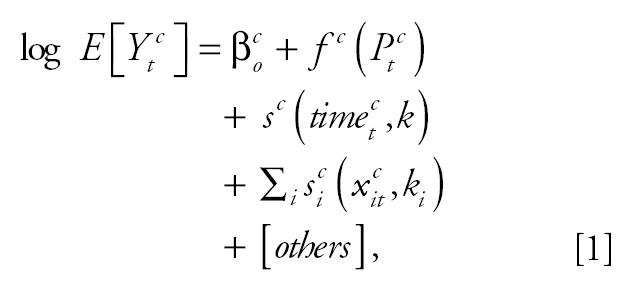


where *E*[*Y*_t_*^c^*] is the expected value of the Poisson distributed variable *Y*_t_*^c^* indicating the count of the health outcome on day *t* at city *c* with var(*Y_t_^c^*) = ϕ*E*[*Y_t_^c^*], ϕ being the overdispersion parameter; *x_it_^c^* is the value of the *x_i_* meteorologic ovariate on day *t* at city *c*;*P*_t_*^c^* is the air pollution level on day *t* at city *c*;*f* *^c^* is the function defining the exposure–response relation between the pollutant and the health outcome; and β*_o_^c^* represents the baseline mortality in city *c*. The smooth functions *s* capture the nonlinear relationship with covariates and can be defined as a linear combination of a set of functions {*b_j_*} with convenient properties; that is, *s* = ∑*_j_a_j_b_j_* (Wood and Augustin 2002). Then *k* is the number of these basis functions {*b_j_*}. We also included dummy variables for the day of the week effect, holidays, and influenza epidemics.

In the last decade, the use of generalized additive models (GAM), which allow non-parametric smooth functions to control for possible confounders, was a standard approach on air pollution time series analysis. Recently, Dominici et al. (2002b) identified that the application of GAM models in the S-Plus software (MathSoft, Inc., Cambridge, MA, USA) with the default convergence criteria leads to biased parameters’ estimates, whereas Ramsay et al. (2003) found in addition that this function underestimated the parameters’ variances. In response to these findings, we used the penalized regression splines as smoothing functions, as implemented by Wood (2000) in R, a public-domain implementation of the S language on which S-Plus is based.

We followed the general methodologic guidelines developed within the framework of the APHEA-2 project, described in detail elsewhere (Touloumi et al. 2004). The basic difference from the APHEA-2 methodology is the use of penalized regression splines instead of the nonparametric function loess as smoothing functions to control for possible confounding. According to the APHEA-2 methodology, these smooth functions of time serve as a proxy for any time-dependent outcome predictors with long-term trends and seasonal patterns not explicitly included in the model. Hence, we remove long-term trends and seasonal patterns from the data to guard against this confounding by omitted variables. Weather variables, which we believe are causally connected to deaths, were also included. In particular, same-day temperature and humidity and a lagged value of these meteorologic variables were also included in the models. We used thin-plate regression splines as basis functions for the penalized regression splines (Wood 2003). In the case of penalized regression splines, as implemented by Wood (2000), *k* in Equation 1 denotes the number of basis functions used for the corresponding variable fit. The choice of a small number of basis functions can have a substantial effect on the final model, because it places an upper bound on how variable the solution can be. Given our experiences from the previous analyses of the APHEA-2 data, we chose the number of basis functions (*k*) to be 40 for the time variable and 10 for the weather variables. We then chose the smoothing parameters that minimized the absolute value of the sum of partial autocorrelations (PACs) of the residuals from lags 3 to 30 days. The choice of lags was based on the fact that in mortality health outcomes there was usually strong remaining PAC in the first two lags of the residuals, which could influence the sum disproportionally. To account for serial correlation in the cases that it remained in the final model residuals, we added autoregressive terms into the model, based on the methodology described by Brumback et al. (2000). In the special case of the small cities (and especially in cause-specific mortality), where the above criterion may lead to almost linear fit for the seasonality, we allowed more degrees of freedom for time provided that this imposed only a minor burden in the sum of the residual PACs. When such a case occurred, we allowed as minimum 1 degree of freedom per year.

Day of the week effects, holidays, and epidemics were controlled for by using dummy variables. We used the APHEA-2 method for influenza control, including a dummy variable taking the value of one when the 7-day moving average of the respiratory mortality was greater than the 90th percentile of its city-specific distribution. Because influenza control as described was based on the distribution of respiratory mortality, we included the influenza dummy variable only when we analyzed total and cardiovascular mortality. Based on previously published results (Braga and Zanobetti 2000; Touloumi et al., In press), there is no indication that omitting control for influenza when we analyzed respiratory mortality would influence the association between air pollution and mortality. It is unclear why the specific time within a winter that an epidemic occurs in a particular city should have much to do with air pollution levels and hence confound the relation under investigation.

Regression cubic splines were used to estimate the exposure–response relationship for each city (Samoli et al. 2003), defined by the function *f* in Equation 1. The regression cubic spline function of a variable *P* is (Durrleman and Simon 1989)


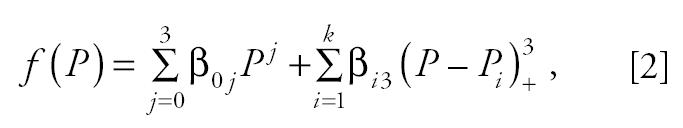


where *k* is the number of knots, and using the + notation of Smith (1979),


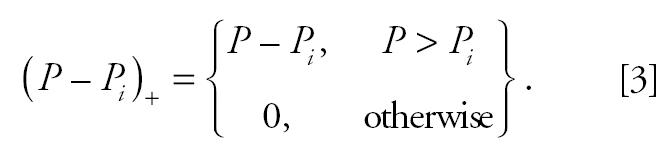


**Figure 1:**
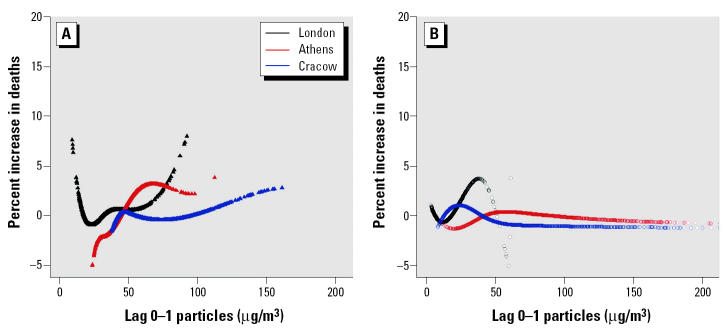
Exposure–response curves of PM_10_ (*A*) and BS (*B*) with total mortality in London, Athens, and Cracow.

For each health outcome, the knots were pre-specified and were the same for each city. This had the advantage that similar terms were pooled in the second stage of the analysis. The number and location of the knots were determined according to exploratory graphical analysis results. Three distinct patterns were dominant across cities in each case—that is, linear and two parabolas. Figure 1 shows the patterns of the particles–total-mortality exposure–response relations in London, England, Athens, Greece, and Cracow, Poland, the largest cities in each of the three distinct geographic areas (western, southern, and eastern European cities). When exploring the PM_10_–mortality relationship we decided to use a cubic spline with two knots at 30 and 50 μg/m^3^, for all mortality outcomes, to sufficiently capture the association in our data. When exploring the relation of BS with mortality we used a regression cubic spline with two knots at 40 and 70 μg/m^3^ in the case of total mortality, at 30 and 60 μg/m^3^ for cardiovascular mortality and at 20 and 50 μg/m^3^ for respiratory mortality.

To further explore indications of potential threshold levels, we fitted threshold models by applying piecewise linear models. We also fitted models with a linear association between the pollutant and mortality to compare the goodness of fit of the different approaches.

### Second-stage analysis.

In the second stage we regressed the city-specific air pollution effect estimates produced form the first stage of the analysis (β*^c^*) on city-specific covariates (**Z***^c^*) to obtain the overall exposure–response (curve and to explore potential heterogeneity in the city-specific curves (Samoli et al. 2003). For the linear model, β*^c^* is the log-relative rate in city *c*, whereas for the spline model, β*^c^* is the vector of the regression coefficients corresponding to the spline function.

For the spline method, we fitted multivariate second stage regression models based on the method described by Berkey et al. (1998). More specifically, the models are of the form


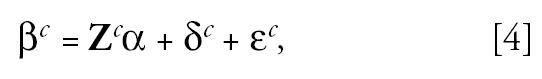


where β*^c^* is the (5 × 1) vector of the five spline estimates in each city *c* (the intercept term in Equation 2 was ignored because only relative risks are considered); **Z***^c^* is a 5 × 5*p* matrix, where *p* is the number of city level covariates for city *c* (including the intercept); α is the vector of regression coefficients to be estimated; δ*^c^* is a vector of five random effects associated with city *c* representing, for each spline estimate, the city’s deviation from the overall model; and ɛ*^c^* (assumed independent from δ*^c^*) is the vector of sampling errors within each city.

The 5 × 5 matrix cov(δ*^c^*) = **D** represents the within-city covariances of the random effects capturing determinants of the city-specific regression coefficients other than sampling error and the city-level covariates considered. It is assumed that δ*^c^* follows the multivariate normal distribution (MVN) with mean **0** and variance-covariance matrix **D**—that is, δ*^c^* ~ MVN (**0**, **D**), and ɛ*^c^* ~ MVN (0, **S***^c^*), β*^c^* ~ MVN (**Z***^c^*α, **D** + **S***^c^*) where **S***^c^* is the covariance matrix of the five regression coefficients of the spline function in city *c* that is estimated in the first stage of the analysis. When **D** ≈ 0 we get the corresponding fixed effects estimates, whereas when **D** ≠ 0 we get the random effects estimates.

The iterative generalized least squares method was applied to estimate model parameters. The parameters of the between-city covariance matrix **D** are estimated by maximum likelihood (Berkey et al. 1998). We applied an overall chi-square test to examine heterogeneity (Touloumi et al. 2004).

When assuming a linear exposure–response relation model, Equation 4 collapses to a univariate one that expresses the usual meta-regression. In this case **D** denotes the between-city variance in the effects estimates and can be estimated from the data using the maximum likelihood method described by Berkey et al. (1995).

After obtaining an overall curve that draws information from all cities, we also compared the two types of models: the linear and the cubic regression spline, within each city and over all cities to determine which best fits the data. We used the Akaike information criterion (AIC) (Akaike 1973) to compare the cubic spline to the linear exposure–response model without threshold representing the standard approach in time series analyses estimating effects of air pollution on mortality or morbidity. For an overall comparison of the different models, we computed the sum of the city-specific AIC values.

As an alternative way to compare the two approaches—the linear and spline models—we computed the difference between the deviances of the fitted models. This difference follows a chi-square distribution with degrees of freedom the difference in the degrees of freedom of the fitted models. For an overall comparison of the different models, we computed the sum of the city-specific differences in deviance, which again follows the chi-square distribution with degrees of freedom the sum of the city-specific difference in the degrees of freedom.

## Results

There was significant heterogeneity for all pollutant–mortality relationships under investigation. Although the observed heterogeneity was either explained or substantially reduced when we investigated the effect modification patterns, all results presented are from the random effects models for consistency reasons. When there was no significant heterogeneity left, results from the fixed-effects models were almost identical to those obtained under the random effects models.

**Figure 2:**
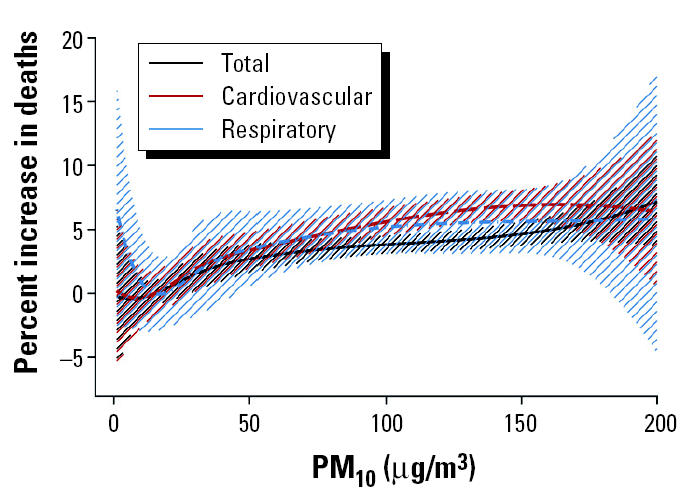
Exposure–response curves and 95% CIs of PM_10_ and total, cardiovascular, and respiratory mortality.

Figure 2 shows the estimated overall exposure–response curves between PM_10_ and total, cardiovascular, and respiratory mortality and their 95% confidence intervals (CIs). Not all cities have values for the pollutant at both ends of the distribution, which is obvious from the wide CIs in the end points of the data. Excluding Stockholm, Sweden, from the analysis, which is the city with the lowest values, the resulting curves were almost identical. Within the range of 36 to 83 μg/m^3^—that is, the common range of the pollutant levels across the analyzed cities—the combined exposure–response curves could be adequately approximated by a linear association. Although all three curves are similar in that range, a steeper slope is indicated for cardiovascular mortality. Overall, for total and cardiovascular mortality, the spline curves are roughly linear, consistent with the absence of a threshold. The curve for respiratory mortality suggests that a threshold model might be reasonable. The downward curve for the exposure–response relationship between respiratory mortality and PM_10_ in the lower end of the distribution of the pollutant is also evident in most of the city-specific exposure–response curves. In the case of total or cardiovascular mortality, this shape is evident in only about five (out of the 22) cities, whereas a linear or logarithmic shape is evident in about half of the analyzed cities. Based on the estimated overall exposure–response curves, an increase from 50 to 60 μg/m^3^ is associated with an increase of about 0.4% in total deaths and with increases of about 0.5% in both cardiovascular and respiratory deaths. These are consistent with the results from regressions assuming a linear relation giving an estimated increase of about 0.5% for total mortality and 0.7% for cardiovascular and respiratory mortality, for a 10-μg/m^3^ increment in PM_10_.

**Figure 3:**
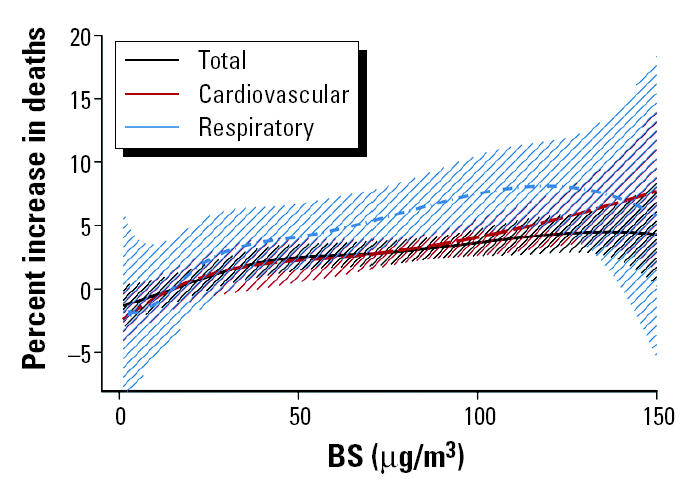
Exposure–response curves and 95% CIs of BS and total, cardiovascular, and respiratory mortality.

Figure 3 shows the estimated combined overall exposure–response curves between BS and total, cardiovascular, and respiratory mortality along with their 95% CIs. As with PM_10_, the spline curves are roughly linear, consistent with the absence of a threshold. In the case of BS, though, the association is steeper between respiratory mortality and the pollutant. This is consistent also with the results assuming a linear association, which indicate a higher increase for respiratory mortality. The bump in the exposure–response relation between respiratory mortality and PM_10_ is not so apparent in the case of BS. Nevertheless, in the lower end of the distribution of the pollutant this association shows a small curvature not observed with the other two outcomes; hence, there is suggestion of a possible threshold.

We examined the hypothesis of linearity in the pollutant–mortality relation more formally by comparing the AIC values obtained under the linear and the spline models. In all cases, both models gave very similar AIC values. Overall the linear model gave a slightly better fit, because the AIC was lower by about 0.1% in all pollutant–mortality combinations. On the other hand, the deviance under the spline model was smaller. In all pollutant–mortality relations, apart from respiratory mortality and BS for which no significant departures from linearity were observed, the overall difference in the deviance between the linear and the spline models was statistically significant, whereas the great majority of the city-specific differences in the deviance of the two models was not statistically significant and in accordance with the findings from the AIC.

We further tested the sensitivity of the results to the number and location of the knots of the spline specification. We re-ran the analysis by specifying one knot at 40 μg/m^3^, and the results were largely similar to the ones presented.

To further explore the indication of a threshold, especially in the case of the association between PM_10_ and respiratory mortality, we applied threshold models with a threshold level at 20 μg/m^3^, because this was indicated by the pooled spline curves. The model comparisons between the linear and the threshold models, based on both the AIC and the difference in the deviance, always chose the linear exposure–response model.

To contribute to the ongoing discussion on whether there is a threshold below current limit values (40 or 50 μg/m^3^), we also fitted threshold models after excluding data at concentrations > 50 μg/m^3^. We tried two threshold models defining the threshold level at 20 and 10 μg/m^3^ because those were indicated by our spline analysis. In any case, the linear models gave a better fit.

We investigated the observed heterogeneity by taking into account the potential effect modifiers through second stage regression models. Potential effect modifiers used in the APHEA-2 analysis included variables describing the air pollution level and mix in each city, the health status of the population, the geographic area, and the climatic conditions (Katsouyanni et al. 2001). We present here the exposure–response curves as shaped by the most important effect modifier from each of the above four distinct categories described above. Namely, we present the associations as they are shaped by the geographic region, the temperature levels, the mean level of nitrogen dioxide (24 hr), and the age-standardized annual mortality rate per 100,000. All the reported effect modifiers were statistically significant apart from the effect of NO_2_ on the association between respiratory mortality and BS. The mean temperature levels in the cities included in the analysis ranged from 6°C in Helsinki, Finland, to 20°C in Tel Aviv, the mean level of NO_2_ (24 hr) ranged from 26 μg/m^3^ in Stockholm to 94 μg/m^3^ in Milan, Italy, and the standardized mortality rate ranged from 430 in Tel Aviv to 1,231 in Lodz, Poland (Table 1). The temperature levels differed significantly among the three geographic areas, whereas the standardized mortality rate differed between the eastern and other cities and the mean NO_2_ 24 hr levels differed between the southern and other cities. The highest correlations (Spearman *r* = 0.86) were observed between temperature and mean NO_2_ 24-hr levels in the cities that provided BS data.

Each of the presented effect modifiers explained in most cases > 20% of the observed heterogeneity. We present the exposure–response curves as observed in the three distinct geographic regions included in the analysis (western cities, southern cities, and central-eastern European cities). We also present the exposure–response curves as shaped for cities with corresponding levels of the presented effect modifier equal to the 25th and the 75th percentile of the distribution of the relevant effect modifier.

**Figure 4:**
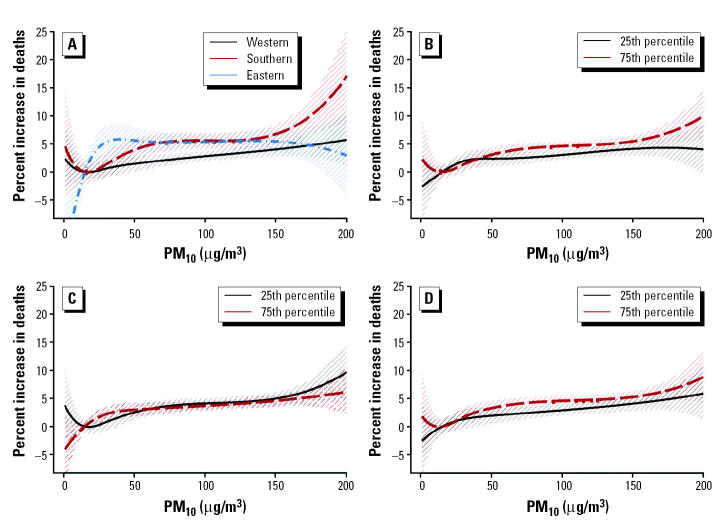
Exposure–response curves and their 95% CIs of PM_10_ and total mortality in different geographic areas (*A*), and in the 25th and 75th percentiles of the distribution of temperature (*B*), standardized mortality rate (*C*), and mean NO_2_ 24-hr levels (*D*).

Figure 4 shows the resulting exposure–response curves (and 95% CIs) for PM_10_ and total mortality. The exposure–response curves for the western and southern cities are similar, although the latter is steeper. The corresponding curve for the eastern cities is very steep in the lower end of the pollutant distribution—that is, at levels < 30 μg/m^3^. However, the minimum value for the pollutant in those areas is 10 μg/m^3^, so in fact the part of the curve below that point is an extrapolation, whereas between 10 and 30 μg/m^3^ only a small proportion of the total data contribute to the estimation, making estimates unstable. The remaining effect modification patterns indicate that the effect of the pollutant on mortality is greater in areas with higher temperature and mean NO_2_ (24-hr) levels, and lower standardized mortality rate. These results are in agreement with those observed when a linear association of PM_10_ and total mortality is assumed (APHEA, unpublished data; Katsouyanni et al. 2001).

When we investigated the heterogeneity of the relation between PM_10_ and respiratory mortality by geographic region, as in total mortality, the exposure–response curves for the western and southern cities were similar, although the latter was steeper. The corresponding curve for the eastern cities had the steepest slope. However, the whole curve was poorly estimated, because of the small counts. The remaining effect modification patterns were not so clear, with lines crossing over the range of the relevant effect modifier. The curve corresponding to the 25th percentile of the NO_2_ (24-hr) distribution is steeper from the level of 50 μg/m^3^ until the level of approximately 150 μg/m^3^, whereas in the range from 20 to 50 μg/m^3^ the slope of the curve corresponding to the 75th percentile is steeper. The curves corresponding to the effect modification by temperature levels are similar, although, as before, in the lower level of the pollutant distribution the slope corresponding to higher temperature is steeper, and in the higher level of the pollutant the slope corresponding to lower temperature is steeper. The effect modification pattern of the standardized mortality rate indicates a steeper slope for higher ratios, except for the range of the pollutant from about 20 to 50 μg/m^3^, where the slope corresponding to lower ratios is steeper.

**Figure 5:**
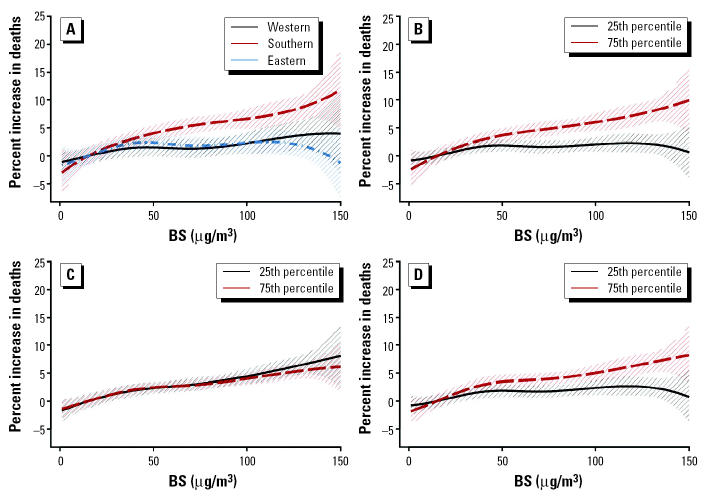
Exposure–response curves and their 95% CIs of BS and total mortality in different geographic areas (*A*), and in the 25th and 75th percentiles of the distribution of temperature (*B*), standardized mortality rate (*C*), and mean NO_2_ 24-hr levels (*D*).

Figure 5 shows the resulting exposure–response curves (and 95% CIs) for BS and total mortality. The effect modification patterns for BS are more linear than the ones observed for PM_10_. Apart from the edges, the exposure–response curves for the western and eastern cities are similar, although the latter is slightly steeper. The corresponding curve for the southern cities indicates the strongest effect of the pollutant on mortality. The other effect modification patterns indicate that the effect of the pollutant on mortality is greater in areas with higher temperature levels and mean NO_2_ (24-hr) levels and lower standardized mortality rates. These results are in agreement with those observed when a linear association of BS and total mortality is assumed (APHEA, unpublished data; Katsouyanni et al. 2001).

When we investigated the heterogeneity in the BS–respiratory mortality association, the curvature observed in the lower end of the overall exposure–response curve of PM_10_ and BS with respiratory mortality (Figure 1) was also apparent in about half of the relationships as those were shaped by the different effect modifiers. As was the case with PM_10_, the exposure–response curve for the eastern cities had the steepest slope. However, also southern cities had, on average, a substantially steeper slope than the western cities, where in fact no relation was observed. The remaining effect modification patterns were not so clear. The curve corresponding to the 25th percentile of the NO_2_ (24-hr) distribution was steeper up to approximately 30 μg/m^3^, and above that the slope of the curve corresponding to the 75th percentile was steeper. Similarly, the curves corresponding to the effect modification by temperature levels indicated that in the lower level of the pollutant distribution the slope corresponding to lower temperature was steeper and in the higher level of the pollutant the slope corresponding to higher temperature was steeper. The effect modification pattern of the standardized mortality rate indicated a steeper slope for higher rates.

## Discussion

In recent years there has been growing demand from policy makers for better understanding of the exposure–response relationship between air pollution and various adverse health effects, including mortality. Most of the relevant studies in Europe were carried out within a small number of locations and consequently have limited statistical power to provide evidence in support of a particular model. We used the most extensive database available in Europe until today (Katsouyanni et al. 2001) to investigate the exposure–response relation between ambient particle concentrations and the daily number of deaths. By use of multiple locations, power is gained and generalizability is enhanced.

We used cubic splines to estimate nonlinear relations of particulate air pollution with mortality. Our results (Figures 2 and 3) indicate that the spline curves for both PM_10_ and BS with total and cardiovascular mortality are roughly linear, consistent with the absence of a threshold. The curve for respiratory mortality suggests that there is some evidence for deviation from linearity in the lowest levels of the pollutants distribution.

There was significant heterogeneity in all associations under investigation. However, the chi-square test applied for the investigation of heterogeneity has very high power when many studies are included in the meta-analysis, and especially when these studies are large, as in our case (Higgins et al. 2003). Formal comparison between spline and linear models based on the AIC indicated that the linear models fit better. The result under the chi-square test indicating that in most of the pollutant–mortality associations the deviance of the spline models is significantly smaller may be an artifact due to the sensitivity of the chi-square test. This claim is supported by the city-specific results, where the conclusions derived from the AIC and the chi-square tests are in agreement. In the great majority of the cities analyzed, the linear and spline models gave very similar fit; hence, the sensitivity of the overall chi-square test picks up the difference in the few other cities. Another possible explanation is that the spline model captures the logarithmic shape of the relation in the higher end of the pollutant’s distribution better, because fitting a logarithmic association with the pollutant gave the best fit.

It is well understood that the measured particle indicators represent a mixture, with varying chemical and physical characteristics, reflected on different toxicity of parts of this mixture. Similarly, the populations studied in our analysis consist of subgroups with different sensitivity to PM exposure. It is likely that the exposure profile and sensitivity of each subgroup (indeed, of each individual) result in various thresholds of effects that cannot be identified with this methodology. The linear curve resulting from our analysis may be seen as a composition of these postulated “partial” curves and may be used effectively for the protection of the whole population. Clearly, more research is needed to identify the most dangerous components of the PM mixture and the most sensitive population subgroups. On the other hand, the biologic mechanisms underlying the PM–health outcome associations are not yet completely clear.

The curvature of the exposure–response relationship between ambient particles and respiratory mortality in the lower levels of the pollutants, not so strongly observed for total and cardiovascular mortality, suggests that there may be different mechanisms underlying the association of particulate pollution exposure to different mortality health outcomes. Goodman et al. (2004) reported a different time response for cardiovascular mortality compared with respiratory mortality, where cardiovascular mortality occurs within the first few days of exposure, whereas respiratory mortality showed a lag of up to 2 weeks. This observed curvature could also be caused by the composition of the air pollution mix at the low concentrations. This rationale is based on the fact that PM_10_ measurements represent all particles with aerodynamic diameter < 10 μm, a mixture of primary and secondary particles from different sources with varying characteristics and levels of toxicity. Unfortunately, the present study does not have enough information to sufficiently investigate this possibility.

Nevertheless, in the range of the pollutants common to all the cities included in the analyses, all associations were approximately linear. The above results are consistent with those reported in previous studies in Europe (Rossi et al. 1999; Schwartz et al. 2001) and in the United States (Daniels et al. 2000; Dominici et al. 2002a; Schwartz and Zanobetti 2000). The slope of the association between ambient particles and total or cardiovascular mortality is higher for levels < 50 μg/m^3^ (and > 10 μg/m^3^ where there is enough information). This is consistent from the results from 10 U.S. cities analyzed by Schwartz (2000b).

Formal comparison between threshold and linear models, based either on the AIC or on the deviance chi-square test, showed that linear models would on average fit better than the threshold ones.

We investigated several factors that potentially influence the exposure–response relations and might provide some explanations for the different shapes observed in different locations. Specifically, in the range of the pollutants common in all analyzed cities, the exposure–response curves between ambient particles and total or cardiovascular mortality were steeper in southern European cities. The association between particles and total and cardiovascular mortality was steeper in locations with hotter climates, higher mean NO_2_ (24-hr) levels, and lower standardized mortality rates. The effect of NO_2_ suggests that particles originating from vehicle exhausts are more toxic than those from other sources. A possible explanation for the temperature effect on the exposure–response association may be that in warmer countries, outdoor fixed-site air pollution measurements may represent the average population exposure better than the measurements in colder climates, because people tend to keep their windows open and spend more time outdoors in warmer climates. Finally, in this study a large age-standardized mortality rate was related to a smaller proportion of elderly persons and probably to the presence of competing risks for the same disease entities. It is therefore related to a smaller proportion of people belonging to vulnerable groups who are more susceptible to air pollution effects. The above-reported effect modification patterns are in accordance with the corresponding ones when a linear pollutant–mortality association was assumed (APHEA, unpublished data; Katsouyanni et al. 2001).

When we investigated the relation with respiratory mortality, the exposure–response curves were steeper in Eastern European cities. The effect modification patterns between ambient particles and respiratory mortality are less clear and need further investigation. In the range of the pollutants common in all analyzed cities, the exposure–response curves are steeper in eastern European cities. Also, in cities with higher standardized mortality rates, the slopes were steeper. These findings supplement each other, because in the cities included in our analysis, all eastern cities had high standardized mortality rates. The effect on the particles–respiratory mortality association of the remaining potential effect modifiers investigated is analogous to the ones observed in the cases of total and cardiovascular mortality. Namely, in the range of the pollutants most commonly observed, cities with higher temperatures and mean NO_2_ (24-hr) levels present steeper slopes.

In conclusion, the association between ambient particles and mortality in the cities included in the present analysis could be adequately estimated using the linear model. Our results confirm those previously reported from Europe and the United States. The heterogeneity found in the different city-specific relations reflects real effect modification, which can be explained partly by factors characterizing the air pollution mix, climate, and the health of the population. Hence, measures that focus on lowering air pollution concentrations have greater public health benefits than those that focus on a few days with the highest concentrations (Clancy et al. 2002). The tendency for a curvature at levels < 20 μg/m^3^, if true, is likely to reflect differences in the mixture and toxicity at different levels. Further study focusing on the composition of particles is needed to further our understanding of the etiologic mechanism through which particles affect mortality and particularly respiratory mortality.

## Figures and Tables

**Table 1:** City descriptive data on the study period, population, exposure (PM_10_ and BS), outcome (daily number of deaths), and selected effect modifiers (region, mean temperature, mean NO_2_ over 24 hr, and directly standardized mortality rate).

			No. of deaths per day			PM_10_ (μg/m^3^) percentile		BS (μg/m^3^) percentile					
City	Study period (month/year)	Population (× 1,000)	Total	CVD	Respiratory	50th	90th	50th	90th	Geographic region	Mean temperature	NO_2_ (24-hr)	SDR
Athens	1/92–12/96	3,073	73	64	5	40*^a^*	59	64	122	South	18	74	784
Barcelona	1/91–12/96	1,644	40	32	4	60	95	39	64	South	16	69	740
Basel	1/90–12/95	360	9	8	1	28*^a^*	55			West	11	38	678
Bilbao	4/92–3/96	667	15	11	1			23	39	South	15	49	711
Birmingham	1/92–12/96	2,300	61	50	9	21	40	11	22	West	10	46	895
Budapest	1/92–12/95	1,931	80	57	3	40*^a^*	52			East	11	76	1,136
Cracow	1/90–12/96	746	18	13	0	54*^a^*	86	36	101	East	8	44	1,009
Dublin	1/90–12/96	482	13	10	2			10	26	West	10	—	940
Erfurt	1/91–12/95	216	6	—	—	48	98			West	9	40	972
Geneva	1/90–12/95	317	6	4	0	33*^a^*	71			West	10	45	608
Helsinki	1/93–12/96	828	18	14	2	23*^a^*	49			West	6	33	915
Ljubljana	1/92–12/96	322	7	5	0			13	42	East	11	46	823
Lodz	1/90–12/96	828	30	20	1			30	77	East	8	39	1,231
London	1/92–12/96	6,905	169	139	29	25	46	11	22	West	12	61	851
Lyon	1/93–12/97	416	9	7	1	39	63			West	12	63	579
Madrid	1/92–12/95	3,012	61	46	6	33	59			South	15	70	636
Marseille	1/90–12/95	855	22	18	2			34	56	West	16	71	666
Milan	1/90–12/96	1,343	29	23	2	47*^a^*	88			West	14	94	632
Netherlands	1/90–9/95	15,400	342	140	29	34	67	63	122	West	10	43	757
Paris	1/92–12/96	6,700	124	91	9	22	46	21	45	West	12	53	644
Poznan	1/90–12/96	582	17	12	1			23	76	East	9	47	1,106
Prague	2/92–12/95	1,213	38	30	1	66	124			East	10	58	984
Rome	1/92–12/96	2,775	56	44	3	57*^a^*	81			South	17	88	585
Stockholm	1/94–12/96	1,126	30	25	3	14	27			West	8	26	666
Tel Aviv	1/93–12/96	1,141	27	22	2	43	75			South	20	70	430
Teplice	1/90–12/97	625	18	13	1	42	83			East	9	32	1,173
Torino	1/90–12/96	926	21	17	1	65*^a^*	129			West	14	76	724
Valencia	1/94–12/96	753	16	14	2			40	70	South	19	66	820
Wroclaw	1/90–12/96	643	15	10	1			33	97	East	9	27	970
Zurich	1/90–12/95	540	13	10	1	28*^a^*	54			West	11	40	666

Abbreviations: —, no data; CVD, cardiovascular deaths; SDR, directly standardized mortality rate. Mean temperature in degrees centigrade.

a PM_10_ were estimated using a regression model relating collocated PM_10_ measurements to the BS or total suspended particles.
